# Analysis of low birth weight and its co-variants in Bangladesh based on a sub-sample from nationally representative survey

**DOI:** 10.1186/s12887-018-1068-0

**Published:** 2018-03-06

**Authors:** Jahidur Rahman Khan, Md. Mazharul Islam, Nabil Awan, Olav Muurlink

**Affiliations:** 1 Institute of Statistical Research and Training, University of Dhaka, Dhaka, Bangladesh; 2 Department of Biostatistics, University of Pittsburgh, Pittsburgh, USA; 3 School of Business and Law, Central Queensland University, Brisbane, Australia; 4 Griffith Institute of Education Research, Nathan, Brisbane, Australia

**Keywords:** Low birth weight, Infants, Rural and urban births, Bangladesh

## Abstract

**Background:**

Low birth weight (LBW) remains a leading global cause of childhood morbidity and mortality. This study leverages a large national survey to determine current prevalence and socioeconomic, demographic and heath related factors associated with LBW in Bangladesh.

**Methods:**

Data from the Multiple Indicator Cluster Survey (MICS) 2012–13 of Bangladesh were analyzed. A total of 2319 women for whom contemporaneous birth weight data was available and who had a live birth in the two years preceding the survey were sampled for this study. However, this analysis only was able to take advantage of 29% of the total sample with 71% missing birth weight for newborns. The indicator, LBW (< 2500 g) of infants, was examined as the outcome variable in association with different socioeconomic, demographic and health-related covariates. Mixed-effects logistic regression was performed to identify possible factors related to LBW.

**Results:**

In the selected sub-sample, about 20% of infants were born with LBW, with lowest rates observed in Rajshahi (11%) and highest rates in Rangpur (28%). Education of mothers (adjusted odds ratio [AOR] 0.52, 95% confidence interval [CI] 0.39–0.68 for secondary or higher educated mother) and poor antenatal care (ANC) (AOR 1.40, 95% CI 1.04–1.90) were associated with LBW after adjusting for mother’s age, parity and cluster effects. Mothers from wealthier families were less likely to give birth to an LBW infant. Further indicators that wealth continues to play a role in LBW were that place of delivery, ANC and delivery assistance by quality health workers were significantly associated with LBW. However there has been a notable fall in LBW prevalence in Bangladesh since the last comparable survey (prevalence 36%), and an evidence of possible elimination of rural/urban disparities.

**Conclusions:**

Low birth weight remains associated with key indicators not just of maternal poverty (notably adequate maternal education) but also markers of structural poverty in health care (notably quality ANC). Results based on this sub-sample indicate LBW is still a public health concern in Bangladesh and an integrated effort from all stakeholders should be continued and interventions based on the study findings should be devised to further reduce the risk of LBW.

## Background

Low birth weight (LBW) remains a leading public health problem especially in developing countries, but in both the developed and developing world LBW remains associated with cardio-metabolic [1, 2] psychiatric disorders [3], and mortality both in infancy [4] and adulthood [2]. It is estimated that between 15% and 20% of all births worldwide are LBW (defined by the World Health Organization (WHO) as < 2500 g) or very low birth weight (< 1500 g), representing a minimum of 20 million infants around the world. The 2500 g cut point is drawn from epidemiologic studies showing that infants with birth weights less than 2500 g are approximately 20 times more likely to die in infancy [1].

The vast majority (95.6%) of LBW births occur in low and middle income countries [5, 6]. In South Asia, the rate of LBW births runs at almost double the global rate [6]. About 70% of all infants with LBW arise in Asia, with central and south Asia showing the highest rates (28%) among all regional zones in the world to experience the problem [6]. The rate of LBW in Bangladesh during the last national survey was high, and arose even in developed urban areas traditionally associated with lower prevalence. The National Low Birth Weight Survey (NLBWS) of Bangladesh (2003–2004) estimated that about 36% of total infants were born with LBW, with 29% prevalence in urban areas [7, 8]. Considering the implications for child mortality [9, 10] significant reduction in prevalence of LBW is necessary to achieve Sustainable Development Goals (SDGs).^1^

Substantial research effort has been expended to assess and identify the determinants of LBW. Findings suggest low birth weight is closely associated in with gestational age, and the two terms are often mistakenly used interchangeably; however, preterm infants (below 37 completed weeks) have a higher mortality weight than full-term infants who are low weight for their gestational age [11]. Preterm birth (short gestation), growth restriction or a combination of both are the main biological causes of LBW, however studies also show significant causal relationships with maternal [12], paternal and passive [13] smoking and drug use, as well as nutritional and micro-nutritional, notably anaemia [14]. Additionally, maternal characteristics including age, [15] maternal anthropometric measurements [16–18] as well as the availability and uptake of ANC facilities [19–21] are commonly associated with LBW. By contrast, in most developing countries, early pregnancy resulting from early marriage is frequently identified as significant causal factor in birth of infants with LBW [8, 19, 22, 23].

In this study, we aim to explore the prevalence statistics on LBW and analyze socioeconomic, demographic and health factors related to LBW in the population of Bangladesh based on a sub-sample from a nationally representative data. Additionally we aim to assess the nation’s progress towards SDGs from this sub-study. This study provides a measure of the success of public health policy and interventions, and at a broad level aims to help shape future approaches to reducing the prevalence of LBW.

## Methods

The data were taken from Bangladesh Multiple Indicator Cluster Survey (MICS) 2012–2013 which was conducted from December 2012 to April 2013 by the Bangladesh Bureau of Statistics (BBS) under the Ministry of Planning [24]. This United Nations Children’s Fund (UNICEF) study helps fill data gaps through household surveys designed to estimate indicators at a national level. The Bangladesh MICS covers urban and rural areas in all sixty-four districts in Bangladesh, under seven administrative divisions. Main objectives of the MICS are to guide policy and intervention by offering a current picture of the welfare of women and children, including maternal and child health. Four sets of questionnaires were administered in the survey. Two of them were used to collect information about children under five years of age (administered to mothers or caregivers) and all women in sampled households aged 15–49 years.

The survey samples were selected using a two-stage stratified cluster sampling procedure. Administrative districts were considered as strata and classified as United Nations Development Assistance Framework (UNDAF) priority districts and non-UNDAF districts. Allocating 20 sample households per cluster, 50 sample clusters were selected from each of 20 UNDAF districts and 40 sample clusters were selected from each of 44 non-UNDAF districts. These sample clusters were selected using the probability-proportional-to-size (PPS) method, based on total number of households in each cluster. The sample households in each cluster were selected from a list of households using a systematic random selection procedure. A total 55,200 sample households in 2760 sample clusters were selected for inclusion. In our study, a sample of 7866 women (15–49 years) who had a live birth in last two years preceding the survey were included. Our analysis focused only on the sub-sample of 2319 mothers, who were able to provide birth weight information.

Outcome variable birth weight was measured in grams categorized as binary variable: *low birth weight* (birth weight < 2500 g) and *normal* (birth weight ≥ 2500 g). Drawing on a range of studies carried out to assess the magnitude of LBW and to identify its determinants [8, 16, 17, 21–23, 25, 26], the following variables were included in the analysis: household wealth, mother’s age in years (“≤20”, “21–30”, “30+”), mother’s education and education of household head, parity (“1”, “> 1”), ANC visit, ANC assistance, delivery assistance, delivered by caesarean, place of delivery and of residence. Levels of household wealth were broken into terciles based on a wealth index created using principal components analysis (PCA) and classified into three groups (“low”, “middle” and “high”). Education level of mother and household head education were each split into two dichotomous variables (“secondary complete or higher”, “others”). The ANC visit variable was coded as “yes” and “no” where ANC assistance was coded as “doctor/nurse/midwife/auxiliary midwife” or “other person”. The classification of place of delivery was “home” and “others” (defined as government hospital, clinic or health facility, or private hospital, clinic, specialist maternity home or other private medical facility), delivery assistance (“doctor/nurse/midwife/auxiliary midwife” and “other person”) and delivered by caesarean (“yes” and “no”). Place of residence was categorized as urban or rural.

We calculated summary statistics of variables, including the prevalence of low birth weight across the socioeconomic, demographic and health related variables. Chi-square tests were performed to find the association between low birth weight and different predictors. We used a mixed effect modelling approach, specifically mixed effects logistic regression, to adjust cluster level variation. Models additionally adjusted for mother’s age, parity. For presentation, we report the adjusted odds ratios (AOR) estimates with 95% confidence intervals (CI) and *p*-values. The analysis was conducted with R (version 3.2.0).

A large number of newborns are delivered at home with no formal record of weight preserved presenting a major challenge in collecting accurate information on weight at birth in developing countries, including Bangladesh. In our dataset, 71% of newborns show no birth weight information. Among the complete cases, there are two sources of information (mother’s recall and health card). We explored the possibility of systematic difference in these two groups, splitting into two sources such as mother’s recall (sample, 1693) and health card (sample, 626) to identify any significant change in the estimates and the association of potential factors related to low birth weight. Most importantly, results based on this sub-sample are not generalizable for overall Bangladesh.

## Results

A total 2319 cases were identified for whom birth weight values were available. Table 1 gives an overview of key descriptive statistics. In our sub-sample, the respondents were largely young, uneducated and rural. Almost half of the selected children were the mother’s first child. About 78% infants were from the rural region of the country, where the highest and the lowest participants were from Dhaka (about 28%) and Sylhet (about 4%) respectively. The majority of mothers visited ANC during their last pregnancy and took assistance from doctor or nurse or midwife during ANC visit. Somewhat surprisingly, one third of children were born at home with 75% cases getting assistance for delivery by doctor or nurse or midwife. About 44% children of selected mothers were delivered by caesarean. Almost all mothers (96%) had exposure to any media (newspapers, radio, and television).

**Table 1:** Summary statistics of selected variables

Variables	Estimates (Total, n = 2319)		
	Frequency, n	Percentage, %	95% CI
Low birth weight			
Yes	469	20.2	18.6–21.9
No	1850	79.8	78.1–81.4
Mother’s age (years)	25.0 ± 5.5 **(**Mean ± SD**)**		
< 20	548	23.6	21.9–25.4
21–30	1429	61.6	59.6–63.6
31+	342	14.75	13.3–16.2
Mother's education			
Secondary complete or Higher	581	25.1	23.3–26.8
Others	1738	74.9	73.2–76.7
Household head education			
Secondary complete or Higher	534	23.1	21.3–24.8
Others	1783	76.9	75.2–78.7
ANC visit			
Yes	2025	87.3	85.9–88.7
No	294	12.7	11.3–14.0
ANC assistance			
Doctor/Nurse/Midwife/Auxiliary midwife	1746	75.3	73.5–77.1
Other person	573	24.7	22.9–26.5
Media exposure			
Yes	2227	96.0	95.2–96.8
No	92	4.0	3.2–4.8
Place of delivery			
Home	763	32.9	30.9–34.8
Others	1556	67.1	65.2–69.0
Delivery assistance			
Doctor/Nurse/Midwife/Auxiliary midwife	1733	74.7	72.9–76.5
Other person	586	25.3	23.5–27.0
Delivered by caesarean			
Yes	1027	44.3	42.3–46.3
No	1292	55.7	53.7–57.7
Parity			
1	1038	44.8	42.7–46.8
> 1	1281	55.2	53.2–57.3
Wealth index			
Low	781	33.7	31.8–35.6
Middle	387	16.7	15.2–18.2
High	1151	49.6	47.6–51.7
Place of residence			
Rural	1807	77.9	76.2–79.6
Urban	512	22.1	20.4–23.8
Division			
Barisal	108	4.6	3.8–5.5
Chittagong	243	10.4	9.2–11.7
Dhaka	651	28.1	26.2–29.9
Khulna	458	19.8	18.1–21.4
Rajshahi	226	9.8	8.5–10.9
Rangpur	536	23.1	21.4–24.8
Sylhet	97	4.2	3.4–5.0

**Fig. 1:**
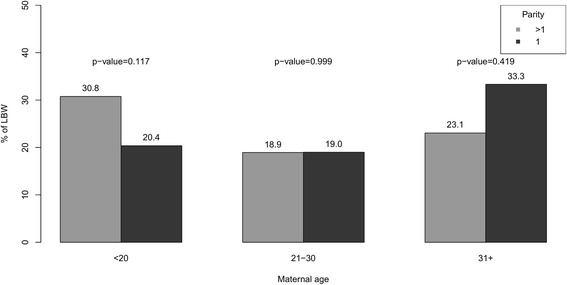
Prevalence of LBW by mother’s age and parity

**Fig. 2:**
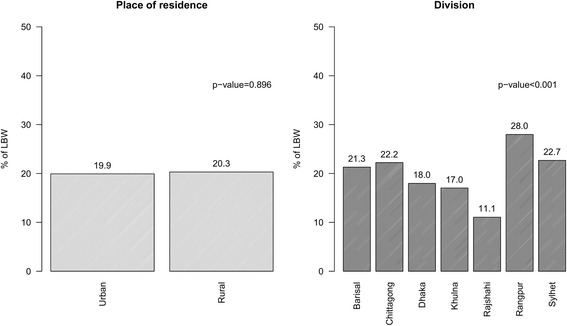
Place of residence and division wise prevalence of LBW

Table 2 reveals that the distribution of low birth weight according to other factors selected for this study. The prevalence of low birth weight was low amongst mothers who had completed at least secondary education or experienced an ANC visit during pregnancy or had a doctor/nurse/midwife/auxiliary midwife in attendance at birth. In addition, the rates of LBW were higher (about 28%) among cases involving delivery at home compared to all other locations. Fewer children delivered by caesarean were of low birth weight, and the prevalence of low birth weight was higher among children from households with low level of wealth than among children from households with mid or high levels of wealth. LBW was notably more prevalent in second or subsequent births amongst young mothers (< 20 years old), whereas the reverse pattern was observed amongst older mothers (31+ years old) (Fig. 1). The prevalence of LBW among infants from rural and urban areas did not differ significantly. LBW varied greatly by geographical division, ranging from about 11% (Rajshahi) to 28% (Rangpur) (Fig. 2).

**Table 2:** Prevalence and Adjusted odds ratio (95% CI) of low birth weight^a^ across covariates

Variables	Total, n = 2319		Card, n = 626		Recall, n = 1693	
	LBW, n (%)	AOR (95% CI)	LBW, n (%)	AOR (95% CI)	LBW, n (%)	AOR (95% CI)
Mother's education (ref.: Others)	391 (22.5)	1.00	124 (27.4)	1.00	267 (20.8)	1.00
Secondary complete or Higher	78 (13.4)	0.52 (0.39–0.68)^***^	22 (12.7)	0.39 (0.23–0.65)^***^	56 (13.7)	0.58(0.41–0.81)^**^
Household head education (ref.: Others)	384 (15.9)	1.00	267 (20.2)	1.00	117 (17.7)	1.00
Secondary complete or Higher	85 (21.5)	0.68 (0.52–0.89)^***^	56 (15.1)	0.64 (0.40–1.01)	29 (25.7)	0.69 (0.49–0.97)^*^
ANC visit (ref.: Yes)	394 (19.5)	1.00	17 (32.1)	1.00	58 (24.1)	1.00
No	75 (25.5)	1.40 (1.04–1.90)^*^	129 (22.5)	1.55 (0.82–2.94)	265 (18.3)	1.41 (0.98–2.02)
ANC assistance (ref.: Other person)	150 (26.2)	1.00	41 (31.1)	1.00	109 (24.7)	1.00
Doctor/Nurse/Midwife/Auxiliary midwife	319 (18.3)	0.63 (0.50–0.80)^***^	105 (21.3)	0.61 (0.39–0.96)^*^	214 (17.1)	0.62 (0.46–0.83)^**^
Place of delivery (ref.: Others)	253 (16.3)	1.00	85 (19.2)	1.00	168 (15.1)	1.00
Home	216 (28.3)	2.13 (2.12–2.14)^***^	61 (33.2)	2.12 (1.37–3.26)^***^	155 (26.8)	2.23 (1.69–2.96)^***^
Delivery assistance (ref.: Other person)	165 (28.2)	1.00	49 (34.5)	1.00	116 (26.1)	1.00
Doctor/Nurse/Midwife/Auxiliary midwife	304 (17.5)	0.52 (0.41–0.66)^***^	97 (20.0)	0.47 (0.30–0.75)^**^	207 (16.6)	0.52 (0.38–0.70)^***^
Delivered by caesarean (ref.: No)	314 (24.3)	1.00	92 (28.3)	1.00	222 (23.0)	1.00
Yes	155 (15.1)	0.54 (0.53–0.54)^***^	54 (17.9)	0.56 (0.37–0.84)^**^	101 (13.9)	0.52 (0.39–0.69)^***^
Wealth index (ref.: Low)	205 (26.4)	1.00	65 (33.7)	1.00	140 (23.8)	1.00
Middle	85 (21.9)	0.78 (0.78–0.79)^***^	28 (25.9)	0.68 (0.40–1.16)	57 (20.4)	0.81 (0.55–1.19)
High	179 (15.6)	0.50 (0.50–0.51)^***^	53 (16.3)	0.38 (0.24–0.59)^***^	126 (15.3)	0.55 (0.41–0.75)^***^
Place of residence (ref.: Urban)	102 (19.9)	1.00	32 (21.5)	1.00	70 (19.3)	1.00
Rural	367 (20.3)	1.02 (0.78–1.34)	114 (23.9)	1.14 (0.72–1.79)	253 (19.0)	0.97 (0.69–1.37)

*p*-value: ^***^ < 0.001, ^**^ < 0.01, ^*^ < 0.05

^a^Models additionally adjusted mother’s age, parity, and conditional on cluster level random effect

Table 2 also shows the association of different socioeconomic and demographic variables with LBW adjusted for maternal age, birth order, and cluster level variation. Children were less likely to be born with LBW to mothers with higher levels of education (AOR 0.52, 95% CI 0.39–0.68) or in households headed by the more educated (AOR 0.68, 95% CI 0.52–0.89). Mothers who did not receive any ANC were 1.40 times more likely to give birth to low weight babies. In addition, the likelihood of low birth weight was lower by about 37% among the mothers who received ANC from doctor/nurse/midwife/auxiliary midwife during last pregnancy. Children who were delivered in home were more likely to be born with low birth weights (AOR: 2.13, 95% CI: 2.12–2.14). In addition, the risk of low birth weight among the children whose mothers received delivery assistance from doctor/nurse/midwife/auxiliary midwife were lower by 48% compared to the others. Moreover, children who were delivered by caesarean were less likely to be born with LBW than other babies (AOR: 0.54, 95% CI: 0.53–0.54). Children were less likely to be born with LBW if they were from the highest tercile of households (households with high levels of wealth) compared to the lowest tercile (households with low levels of wealth). Results confirmed that the risk of LBW did not differ significantly among rural and urban population.

The separate analyses for card and recall birth weight data are presented in Table 2. Although there were some differences in the point estimates between card and recall data, the confidence intervals were overlapping.

## Discussion

Low birth weight remains one of the major public health challenges in Bangladesh. Our findings based on sub-sample reveal that about 20% of the children are born as low birth weight babies, which is consistent with the reported prevalence of low birth weight (22 %) in Bangladesh [27]. However, this represents a significant decline in the rate of LBW since the last comparable national study [7]. Our sub-sample based estimate of prevalence may not be an accurate estimate of the population prevalence due to the large amount of missing information and this is not the prime focus of this study. Instead, this study identifies key variables associated with LBW, besides regional variation. Our analysis demonstrates that the higher maternal education, presence of formal ANC, delivery in a non-home setting, delivery by a health professional or para-professional, delivery by caesarean, and a higher wealth index have statistically significant lower risk of low birth weight infants. In addition, our comparison of two sources of birth weight data indicates that maternal weight recall is an accurate indicator of actual birth weight, a finding that has implications for future research in developing world contexts.

The key finding of an association between maternal and household head secondary education and low birth weight is in accord with previous research, but may be as much an indicator of an association between wealth and associated access to adequate nutrition, as it is between education and access to information about proper family planning and maternal feeding practices [28–31].

This sub-sample based study found a significant association between ANC and low birth weight, with mothers who had access to ANC during pregnancy having significantly lower risk of bearing a LBW child. This is consistent with different studies done in Ethiopia and Nepal [32, 33], but the mediating variable may again be poverty. ANC services generally provide regular monitoring of height-weight gain, diagnosing maternal or foetal problems and thus allowing early intervention and nutritional supplementation which may reduce adverse pregnancy outcomes including LBW [34]. Nutritional supplement programs by non-government organizations may arrest or reverse otherwise likely low birth weight outcomes. Moreover, the *quality* of ANC received by women was also found to be critical [35, 36]. The risk of LBW was lower among the women who received ANC assistance from doctor/nurse/midwife/auxiliary midwife. Again the quality of care received may be determined by ability to pay or location in a region with more advanced health infrastructure. Optimum utilization of ANC services should be further investigated to understand barriers as well as opportunities to improve services in community level.

The proportion of LBW newborns was significantly higher for mothers who delivered at home, a finding is in accordance with studies conducted in India [37, 38]. Our study illustrates that, mothers who received skilled attendance of health workers during birth at home were less likely to deliver LBW children. Skilled attendance at childbirth may reduce low birth weight.

One finding clearly at variance with international trends is the finding that children delivered by caesarean were less likely to be of low birth weight. This may be due to the fact that relatively advanced interventions are more accessible to wealthier households. Certainly, our study also showed that children from wealthy households were less likely to be low birth weight, in line with international studies [23, 39, 40]. This finding needs to be treated with caution, however, since the most recent National Low Birth Weight Survey (NLBWS) of Bangladesh 2015 shows that the odds of LBW are 28% lower for caesarean section as compared to normal [41]. This could be related to the alarming rise in the incidence of caesarean operations over time (from 3.7% in 2003–04 to 35.5% in 2015) [41].

Contrary to previous epidemiological studies of Bangladesh, our study finds a possible evidence that previously stark differences in birth weights between children born in rural and urban areas has been eliminated, with a particularly dramatic fall in LBW prevalence in rural areas largely explaining this change. The most recent National Low Birth Weight Survey of Bangladesh (2003–2004) estimated that about 36% of total infants were born with LBW, with 29% prevalence in urban areas and 37% in rural areas. Our figures based on sub-sample suggest that both figures have now dropped to around 20%, perhaps indicating that rural-urban disparities in LBW prevalence have been improved in the Bangladesh MICS 2012–13. Although given that the frequency of children being weighed at birth varies significantly between urban and rural areas [24], and that our study might be limited by potential selection bias, conclusions about rural-urban disparities may not be generalized based on these data.

To reduce the prevalence of LBW and to improve the conditions of the discussed risk factors, interventions need to be accelerated at multiple levels such as country/region (e.g. to ensure women’s educational attainment and empowerment, social protection systems for improving health-care visits, ensure the consumption of adequately iodized, improvement in facility-based perinatal care in low coverage regions etc.). Community-level interventions (e.g. adequate nutrition for adolescent girls, community-based packages of care to improve linkage and referral for facility births, intermittent iron and folic acid (IFA) supplements for women of reproductive age and adolescent girls due to the high prevalence of anaemia etc.) are also suggested. Interventions relating to planning prior to pregnancy (e.g. planning appropriate birth spacing and peri-conceptional daily IFA supplementation for reduction of congenital anomalies), and antenatal care (e.g. fetal growth monitoring and neonatal size evaluation at all levels of care, ensure daily IFA supplements during pregnancy, decrease in non-medically indicated caesarean delivery and induction, postnatal care interventions to all women, early initiation and promotion of exclusive breastfeeding at community and facility level, balanced protein-energy supplementation, daily calcium supplementation for women in settings with low calcium intake, progesterone therapy for women at risk of preterm birth) are also recommended [6]. Regular replications of LBW surveys to measure the progress towards the reduction of LBW in Bangladesh should be carried out.

### Limitations

A number of limitations suggest that the findings need to be treated with caution. First of all, some selection bias is likely to have arisen because of the large number of cases with missing data relating to birth weight of infants, with almost 71% of infants were not weighed at birth. Due to this, it is probable that the overall prevalence of LBW is underestimated or over estimated. Moreover, children who were not weighed at birth were more likely to be born of older (about 75% in the maternal age group 35–49 years), comparatively less-educated mothers (about 80% to non-educated mothers) and belong to households with low level of wealth (about 77% born to the households with low level of wealth) and rural region (about 69%) than children who were weighed at birth [24]. Exclusion of these children, who are also more likely to have LBW biases the observed associations between these variables and LBW. Secondly, use of mother’s self-reported data (recall) should be noted as one of the limitations; however, it is worth noting that even developed-world studies frequently rely on recalled birth weight [3]. The fact that we found birth weight reported by maternal recall to have similar patterns compared with objectively measured birth weight from the health cards suggests this is not a significant source of error.

## Conclusions

While there was significant erosion in sample size because of large number of missing data, the conclusion that there has been a significant drop in prevalence of LBW births is supported by a later study on birth weights, done at a large single medical college hospital in Dhaka in 2003–2005. Being an urban study, our data (and international studies) would suggest this study [8] would be biased towards fewer low birth weights but the current study shows that LBW rates in non-home settings has fallen to 16.3% (combining rural and urban births), compared to this study’s hospital birth rate of 23.2%. Thus, the current sub-sample based study does provide strong evidence that there has been a significant drop in the prevalence of LBW births in Bangladesh in the last decade, with an additional elimination of the previous large disparity between rural and urban births. It further indicates that use of maternal self-reports for birth weights is an adequate proxy for actual birth weights in developing world epidemiological studies. It confirms that maternal socioeconomic status, ANC received, place of delivery, delivery assistance are important covariates of LBW in Bangladesh, and economic progress, associated with an increase in educational status of women remains a priority in tackling the prevalence of low birth weight. Moreover, integrated and complementary strategies, as well as effective and efficient interventions based on this study finding, are needed to reduce low birth weight among infants to ensure the potential threat of LBW to the growth, health, and survival of both children and adults in Bangladesh.

## Abbreviations

ANC: Antenatal Care
AOR: Adjusted Odds Ratio
BBS: Bangladesh Bureau of Statistics
CI: Confidence Interval
IFA: Iron & Folic Acid
LBW: Low Birth Weight
MICS: Multiple Indicator Cluster Survey
NLBWS: National Low Birth Weight Survey
PCA: Principal Components Analysis
PPS: Probability Proportional to Size
SD: Standard Deviation
SDG: Sustainable Development Goal
UNDAF: United Nations Development Assistance Framework
UNICEF: United Nations Children’s Fund
WHO: World Health Organization

## Footnotes

1 In 2012, member states of the World Health Assembly (WHA) resolved to aim for a 30% reduction in the rate of infants born with low birth weight by 2025.
